# Isolation, Identification, and Characterization of Bioactive Peptides in Human Bone Cells from Tortoiseshell and Deer Antler Gelatin

**DOI:** 10.3390/ijms24021759

**Published:** 2023-01-16

**Authors:** Tsung-Jung Ho, Jung-Hsing Lin, Shinn Zong Lin, Wan-Ting Tsai, Jia-Ru Wu, Hao-Ping Chen

**Affiliations:** 1 Integration Center of Traditional Chinese and Modern Medicine, Hualien Tzu Chi Hospital, Hualien 970473, Taiwan; 2 Department of Chinese Medicine, Hualien Tzu Chi Hospital, Hualien 970473, Taiwan; 3 School of Post-Baccalaureate Chinese Medicine, Tzu Chi University, Hualien 970473, Taiwan; 4 Department of Biochemistry, School of Medicine, Tzu Chi University, Hualien 970374, Taiwan; 5 Department of Neurosurgery, Hualien Tzu Chi Hospital, Hualien 970473, Taiwan

**Keywords:** deer antler, Guilu Erxian Jiao, kyotorphin, neokyotorphin, osteoblast

## Abstract

Tortoiseshell and deer antler gelatin has been used to treat bone diseases in Chinese society. A pepsin-digested gelatin peptide with osteoblast-proliferation-stimulating properties was identified via LC-MS/MS. The resulting pentapeptide, TSKYR, was presumably subjected to further degradation into TSKY, TSK, and YR fragments in the small intestine. The above four peptides were chemically synthesized. Treatment of tripeptide TSK can lead to a significant 30- and 50-fold increase in the mineralized nodule area and density in osteoblast cells and a 47.5% increase in the number of chondrocyte cells. The calcium content in tortoiseshell was relatively higher than in human soft tissue. The synergistic effects of calcium ions and the peptides were observed for changes in osteoblast proliferation and differentiation. Moreover, these peptides can enhance the expression of RUNX2, OCN, FGFR2, and FRFR3 genes in osteoblasts, and aggrecan and collagen type II in chondrocyte (patent pending).

## 1. Introduction

Osteoarthritis is a common bone disease that typically presents in the elderly population. Chondroitin sulfate is a dietary supplement that is predominantly used in the treatment of osteoarthritis, with a global market size worth approximately USD 1.17 billion in 2020; however, its efficacy remains controversial [1]. Tortoiseshell and deer antler gelatin (also known as Guilu Erxian Jiao) have been used for the treatment of bone diseases, such as degenerative joint disease, osteoporosis, osteoarthritis, and joint pain in traditional Chinese medicine for hundreds of years [2,3,4]. This preparation was first named in the *Investigations of Medical Formulas (*醫方考*)*, an ancient Chinese medical book published in 1584 B.C. The formulation often includes tortoiseshell, deer antler, goji berry, and ginseng.

The importance of calcium in human bones has been well documented. Calcium absorption is regulated by many factors, including estrogen, fibroblast growth factor 23, parathyroid hormone, insulin-like growth factor 1, and calcitriol (vitamin D) [5]. Both tortoiseshell and deer antler are derived from hard animal tissue composed of organic and inorganic materials, notably calcium ion [6]. The calcium content in deer antlers and tortoiseshell is about 0.19% and 0.18%, respectively. Therefore, this gelatin can provide a rich source of calcium to patients. On the basis of the efficacy of the tortoiseshell and deer antler gelatin in human bone disease, it is reasonable to propose that the organic components in tortoiseshell and deer antler gelatin could enhance the bioavailability of calcium in human bone systems. This might be the reason why this gelatin is used to treat osteoporosis. On the other hand, the breakdown of cartilage in the joints and changes in the bones of the joints, including bone spur formation, lead to osteoarthritis, causing pain and inflammation. Therefore, in this study, we used human osteoblast and chondrocyte cell lines for in vitro activity assay. Finally, the aim of this study was to isolate and identify the organic components that may stimulate or enhance the activity of human bone cells via treatment with this gelatin.

## 2. Results

### 2.1. Sample Preparation, Separation, and Identification

In traditional Chinese medicine, the original formula for “tortoiseshell and deer antler gelatin” includes tortoiseshell, deer antler, goji berry, and ginseng. However, the herbal components, goji berry and ginseng, were not thought to have any specific effect in bone disease treatment. To facilitate the isolation task for the organic compounds that may actually enhance human calcium bioavailability, a commercial product containing tortoiseshell (from *Mauremys reevesii*) and deer antler (from *Cervus elaphus*) only was obtained from a GMP pharmaceutical company for this study. Both the antler of red deer and tortoiseshell are keratinous hard tissues [6,7]. “Tortoiseshell and deer antler gelatin” was boiled for over 72 h during the preparation process. The proteins within the sample are subject to random breakdown into smaller peptides. This traditional medicine is generally administered orally, passing through the gastrointestinal tract; pepsin is thought to be the first protease to cleave the proteins or peptides in the gastrointestinal tract.

**Figure 1:**
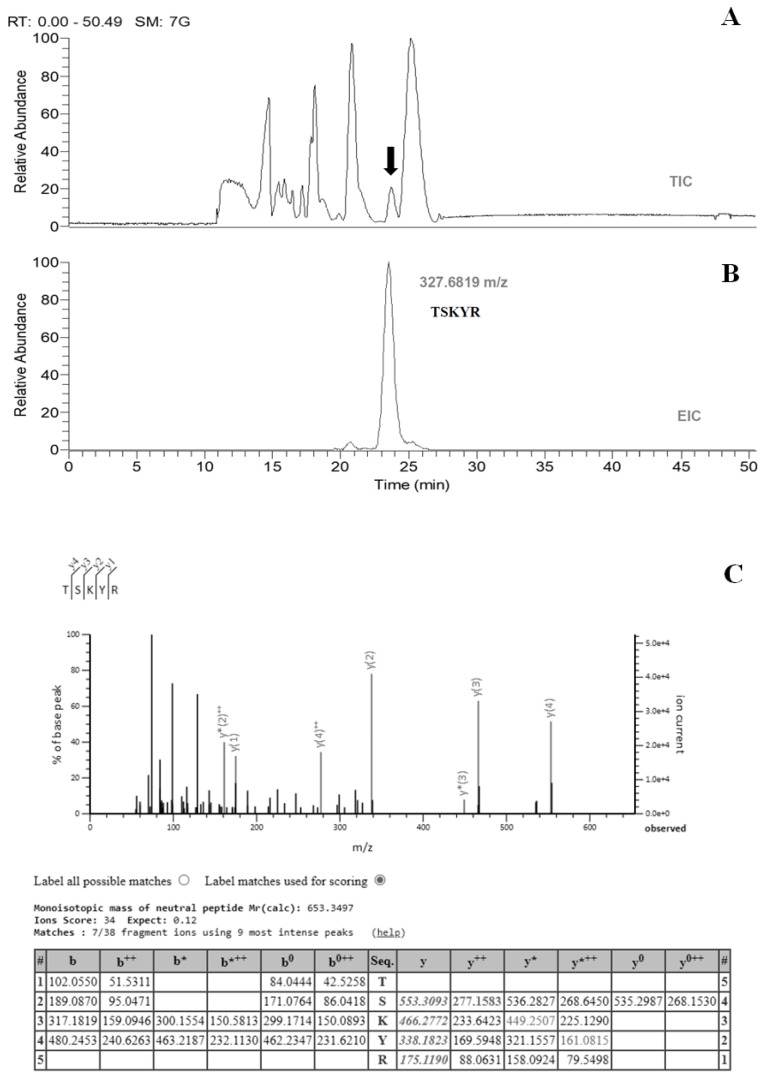
Identification of active peptide from pepsin-digested samples by LC-MS. (**A**) Total ion chromatogram (TIC) of test sample, where the vertical arrow indicates the elution peak with the activity of stimulating osteoblast proliferation. (**B**) Extracted chromatogram (EIC) of test sample, the identified peak with a *m*/*z* value of 327.6819. The values of observed and calculated molecular weights were 653.3493 and 653.3497, respectively (mass error = −0.47 ppm). (**C**) Collision-induced dissociation (CID) spectrum of the active peptide.

The pepsin-digested sample was initially separated by reverse-phase HPLC. The osteoblast proliferation activities were stimulated, and the collected fractions were measured as described in Section 2.5. The active fractions were added together and dried with a recovery rate of 30.8%. The collected samples were subjected to LC-MS/MS analysis directly using different HPLC solvents and elution programs. The active compound was eluted in a well-resolved single peak (Figure 1A), in order to identify the single peak with activity at *m*/*z* = 327.6819 (Figure 1B). The peptide sequence TSKYR was determined by MS/MS fragmentation (Figure 1C), which also appears in the *Cervus elaphus* (red deer) UniProt protein database.

“Tortoiseshell and deer antler gelatin” is taken via the enteral route and is digested by many proteases in the human gastrointestinal tract. It is reasonable to assume that the pepsin-digested pentapeptide TSKYR may be further degraded into smaller fragments during the digestion process. Considering the specificity of chymotrypsin and trypsin present in the small intestine, we examined the activity of the following four peptides: pentapeptide (peptide 5, P5), TSKYR; tetrapeptide (peptide 4, P4), TSKY; tripeptide (peptide 3, P3), TSK; dipeptide (peptide 2, P2), YR.

### 2.2. Measurements of Osteoblast Proliferation-Stimulating Effect

**Figure 2:**
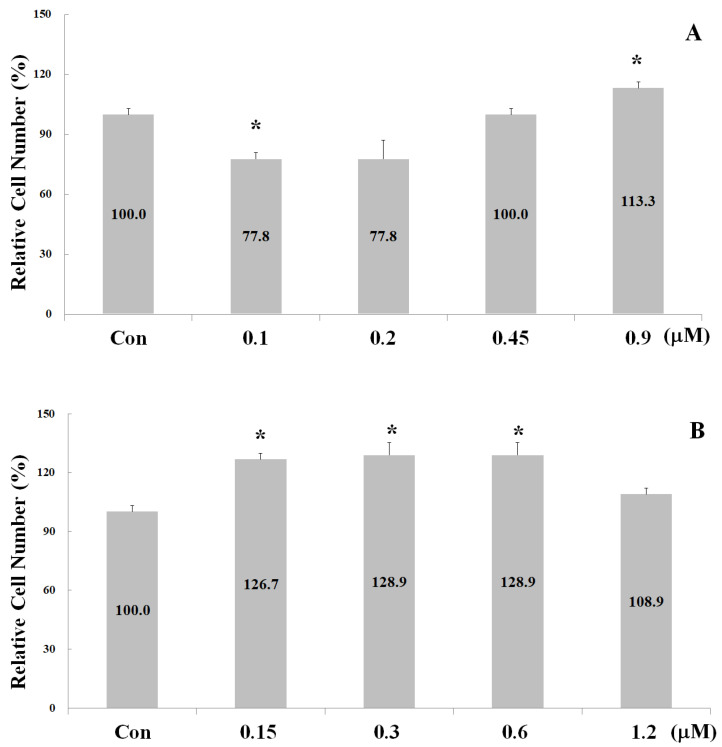

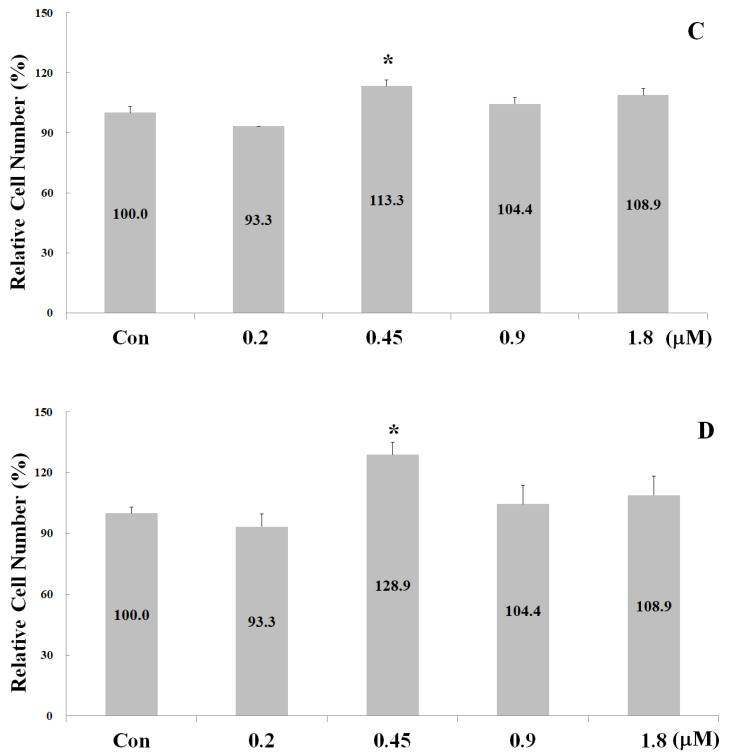
Effects of the synthetic peptides on osteoblast proliferation after treatment for 24 h. (**A**) Peptide 5, (**B**) Peptide 4, (**C**) Peptide 3, and (**D**) Peptide 2. Note: Data are presented as means ± SD in three independent experiments. The differences between the various peptide concentration treatments and the control groups (**A**–**D**) were analyzed using a one-tailed test, * *p* < 0.05.

**Figure 3:**
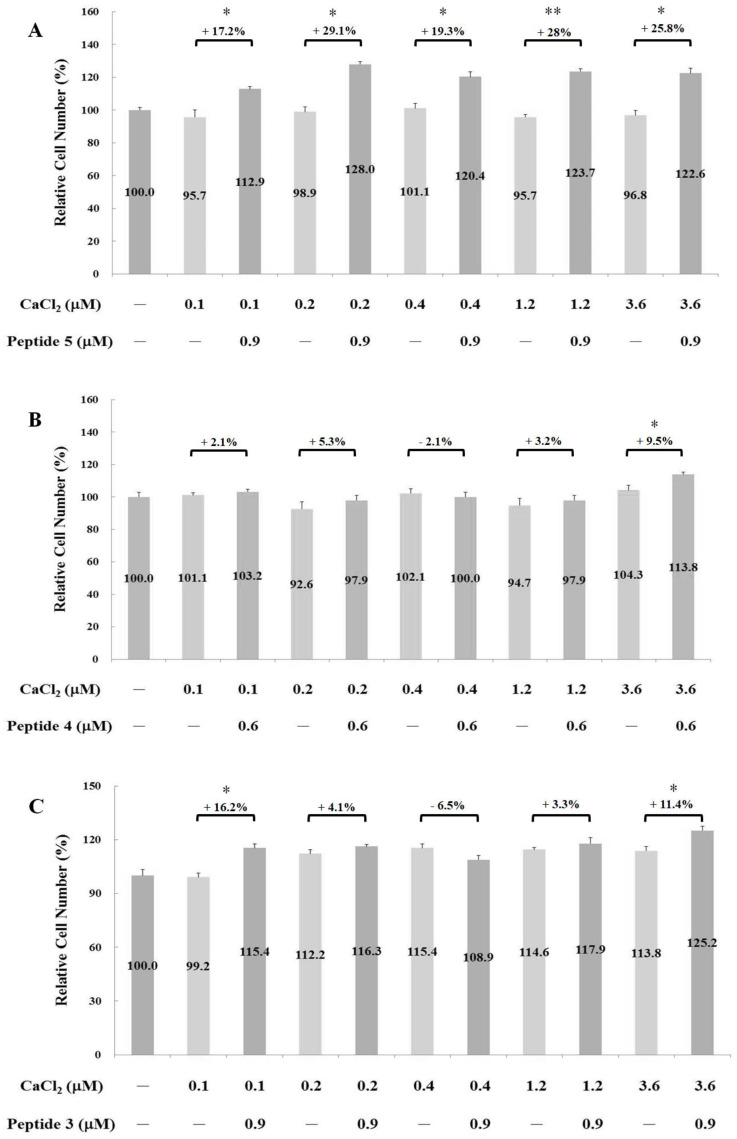

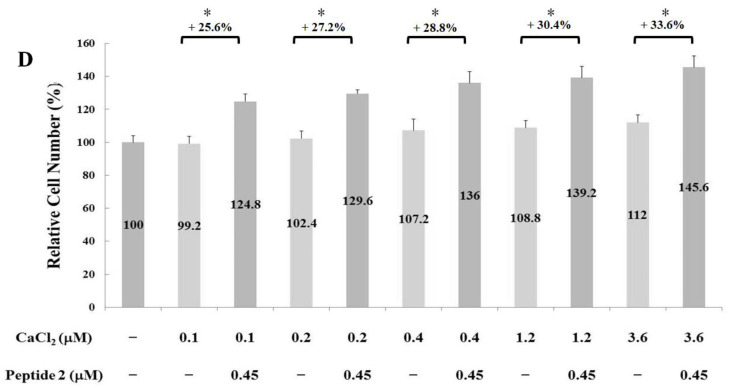
Synergistic effects of calcium ions with each of the four peptides. Note: Osteoblast proliferation activity was represented as the cell number. Data are presented as means ± SD in three independent experiments. The differences between the peptide combined with CaCl_2_ treatment and the only CaCl_2_ treatment groups (**A**–**D**) were analyzed using a one-tailed test, * *p* < 0.05 and ** *p* < 0.005.

The proliferation-stimulating effects of the four synthetic peptides on human osteoblast hFOB1.19 were evaluated. The hFOB1.19 cell numbers were manually counted after treatment with their respective peptides for 24 h. As shown in Figure 2, the optimal working concentrations for peptides 5, 4, 3, and 2 were 0.9, 0.6, 0.45, and 0.45 µM, respectively. Calcium ions alone can trigger critical osteoblast proliferation and differentiation signals during bone remodeling [8]. To further elucidate the synergistic interactions between calcium ions and the above peptides, cell-proliferation experiments were carried out in the presence of 0.1, 0.2, 0.4, 1.2, and 3.6 µM CaCl_2_. As shown in Figure 3, the addition of peptides 5, 4, 3, and 2 led to a cell-number increase of 29.1% (with 0.2 µM CaCl_2_), 9.5% (with 3.6 µM CaCl_2_), 16.2% (with 0.1 µM CaCl_2_), and 33.6% (with 3.6 µM CaCl_2_), respectively. These results clearly represent the synergistic effect of calcium ions on peptides 5, 3, and 2. The strength of the proliferation-stimulating effect on osteoblast hFOB1.19 was in the order of peptide 2 > peptide 5 > peptide 3 > peptide 4. 

### 2.3. Measurement of Chondrocyte Proliferation-Stimulating Effect

**Figure 4:**
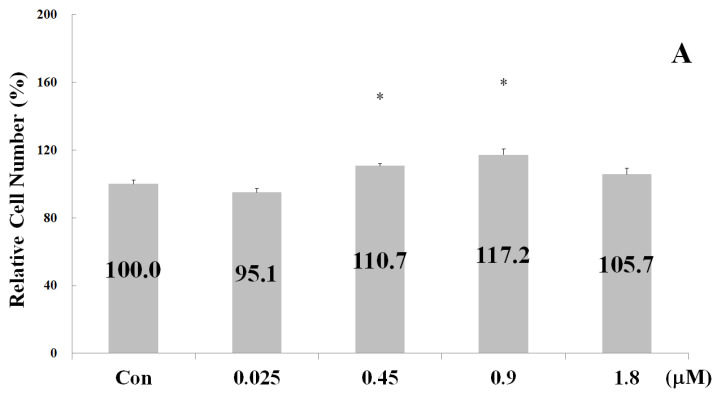

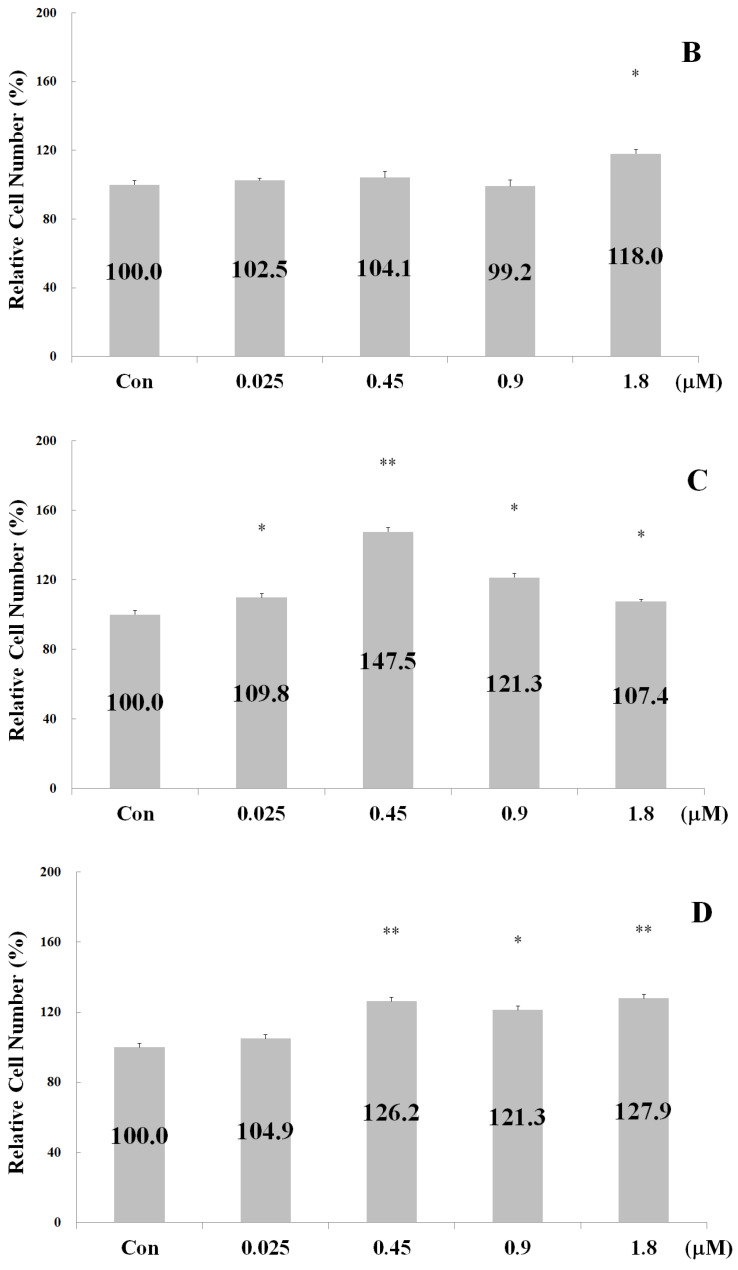
Effects of the synthetic peptides on chondrocyte proliferation after treatment for 24 h. (**A**) Peptide 5; (**B**) Peptide 4; (**C**) Peptide 3; (**D**) Peptide 2. Note: Chondrocyte proliferation activity was represented as the cell number. Data are presented as means ± SD in three independent experiments. The differences between the various peptide concentration treatment and the control groups (**A**–**D**) were analyzed using a one-tailed test, * *p* < 0.05 and ** *p* < 0.005.

The proliferation-stimulating effect of the four synthetic peptides on chondrocyte C20A4 was also measured. Cells were treated with various concentrations of synthetic peptides for 24 h. As shown in Figure 4, the optimal proliferation-stimulating concentrations for peptides 5, 4, 3, and 2 were 0.9, 1.8, 0.45, and 1.8 µM, respectively. Accordingly, the inclusion of peptides 5, 4, 3, and 2 led to a 17.2%, 18.0%, 47.5%, and 27.9% corresponding increase in the number of chondrocyte C20A4 cells. Therefore, the strength of the proliferation-stimulating effect on chondrocyte C20A4 was in the order of peptide 3 > peptide 2 > peptide 4 > peptide 5. These results indicate that the proliferation-stimulating effect of the peptides on osteoblasts and chondrocytes is different. Notably, in contrast with osteoblast hFOB1.19 cells, calcium ions did not promote a proliferation-stimulating effect on chondrocyte C20A4 cells.

### 2.4. Alkaline Phosphatase Activity

**Figure 5:**
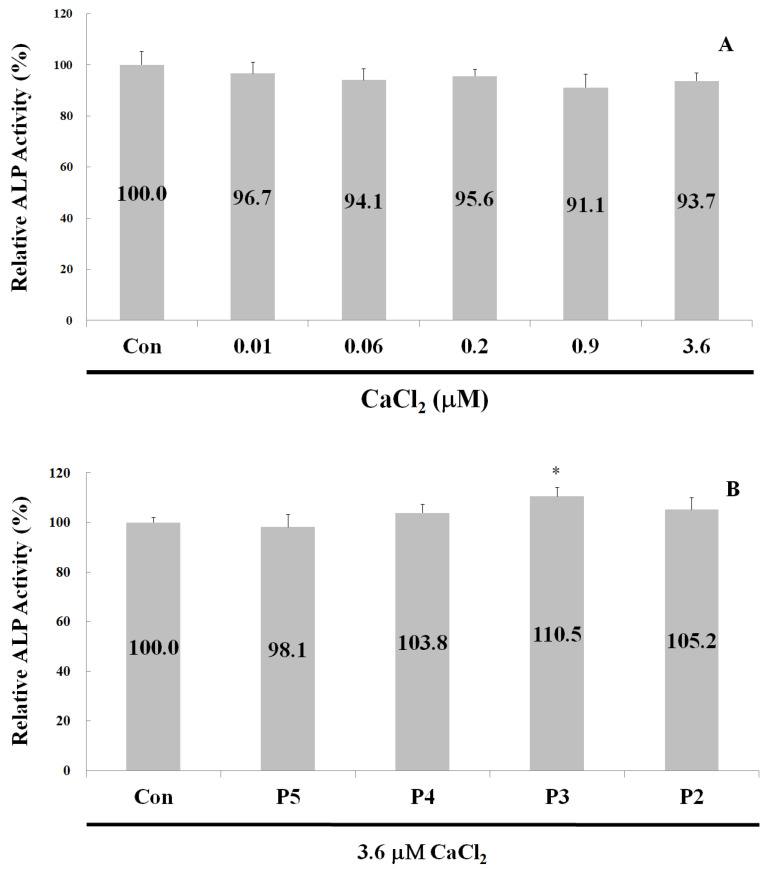
Alkaline phosphatase activity of osteoblasts hFOB1.19 after two days of treatment. (**A**) Treatment with various concentrations of CaCl_2_ only. (**B**) Treatment with 0.45 µM synthetic peptides and 3.6 µM CaCl_2_. P5 = peptide 5; P4 = peptide 4; P3 = peptide 3; and P2 = peptide 2. Data are presented as means ± SD in three independent experiments. The differences between the various peptide treatment and the control groups were analyzed using a one-tailed test, * *p* < 0.05.

ALP is usually used as a marker of osteoblast differentiation and a sign of bone formation [9]. The ALP activities of osteoblast hFOB1.19 cells were examined for 2 d after treatment with 0.45 µM peptides 5, 4, 3, and 2 in the presence of 3.6 µM CaCl_2_. As shown in Figure 5A, no significant changes in the ALP activity of osteoblasts were observed after treatment with 0.01, 0.06, 0.2, 0.9, and 3.6 µM CaCl_2_. However, the addition of 0.45 µM peptides 4, 3, and 2 led to a 3.8%, 10.5%, and 5.2% increase in ALP activity in the presence of 3.6 µM CaCl_2_ (Figure 5B). Thus, a synergistic effect between calcium ions and peptides 4, 3, and 2 was shown to promote osteoblast differentiation and bone formation by stimulating osteoblast proliferation.

### 2.5. Mineralized Nodule Formation

**Figure 6:**
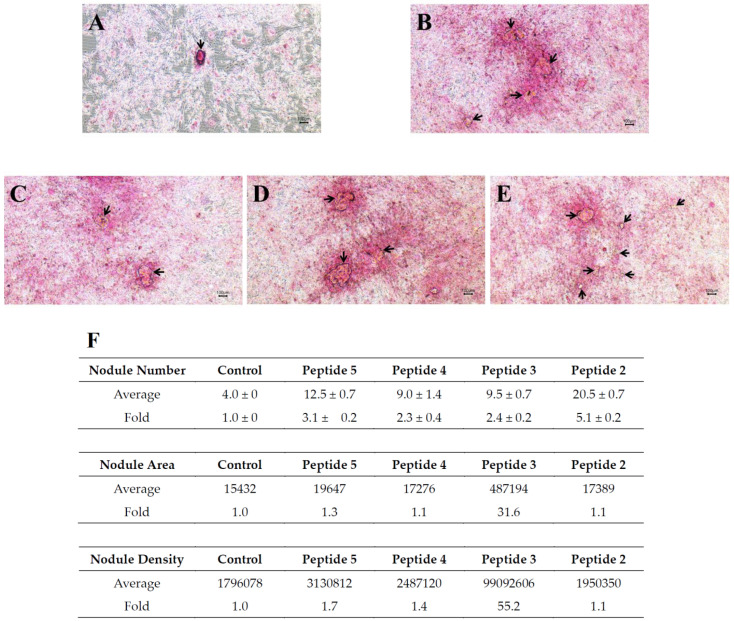
Representative images of Von Kossa staining showing mineralization ability on osteoblast hFOB1.1 cells after the treatment with the synthetic peptides. (**A**): Control, (**B**): peptide 5, (**C**): peptide 4, (**D**): peptide 3, (**E**): peptide 2. Black arrows show mineralized nodules (light microscopy, 40×), (**F**): quantitative analysis of mineralized bone nodule formation using image analysis software. The units for nodule area are pixels and units for nodule density are in arbitrary units (AU). Average number of nodules, average nodule area, and average nodule density were shown.

The number of total calcified nodules is a specific marker for indicating the maturation of cultured osteoblasts. A Von Kossa stain was used to evaluate mineralized nodule formation after treatment with 0.45 µM synthetic peptides. The calcified nodules in hFOB1.19 were stained at 21 d. As shown in Figure 6A–E, mineralized nodules were visible as brown or black colored spots, as indicated by the arrows. In contrast, the background is light pink. When compared to the control group, there was a significant increase in mineralized nodules observed in the peptide treatment groups.

The relative number of mineralized nodules was also calculated (Figure 6F). The treatment of peptide 5, 4, 3, and 2 resulted in a 3.1-, 2.3-, 2.4-, and 5.1-fold increase in the number of mineralized nodules, respectively. Evidently, peptide 2 was most effective at promoting mineralized nodule deposition among the four tested peptides. The area and density of nodules were also measured for each group. Markedly, treatment with peptide 3 led to a substantial 31.6-fold increase in nodule area and a 55.2-fold increase in nodule density. In conclusion, whilst treatment with all four synthetic peptides can increase the number of calcified nodules, peptide 3 treatment was the most effective at enhancing the density and size of the nodules.

### 2.6. Gene Expression

RUNX2 is necessary for osteoblast proliferation, regulating FGFR2 and FGFR3 [10,11,12]. RUNX2 and OCN are the genes involved in the regulation of osteogenic proliferation and differentiation [13], and they are considered to be osteogenic markers. In addition, Wnt, bone morphometric protein, fibroblast growth factor, insulin-like growth factor-1, Hedgehog, and Notch signaling pathways play important roles during osteogenesis and bone repair [14,15,16]. Notably, the members of the TGF-β superfamily regulate the proliferation and differentiation of bone-related cells [17,18,19]. The abnormal mutation generally leads to various bone disorders [20]. To investigate the osteogenesis and chondrogenesis mechanism induced by these peptides, quantitative PCR was carried out to examine the expression of possible genes after treatment of osteoblasts hFOB1.19 cells and chondrocytes C20A4 cells with 0.9 µM of peptide 5, 0.45 µM of peptide 3, and 0.45 µM of peptide 2, with or without the treatment of 18.4 µM LY364947 and 15 µM PQ.

**Figure 7:**
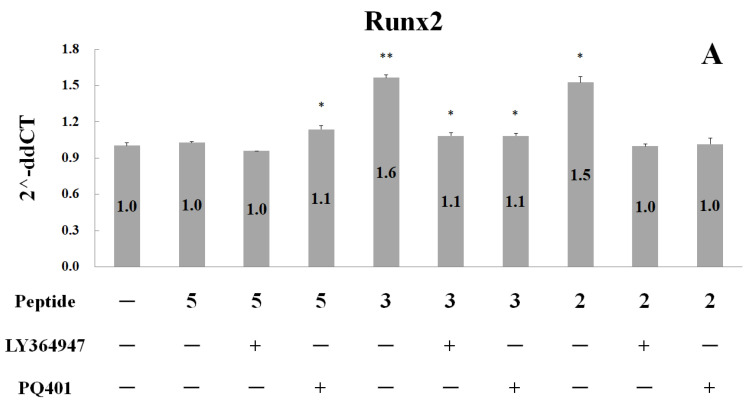

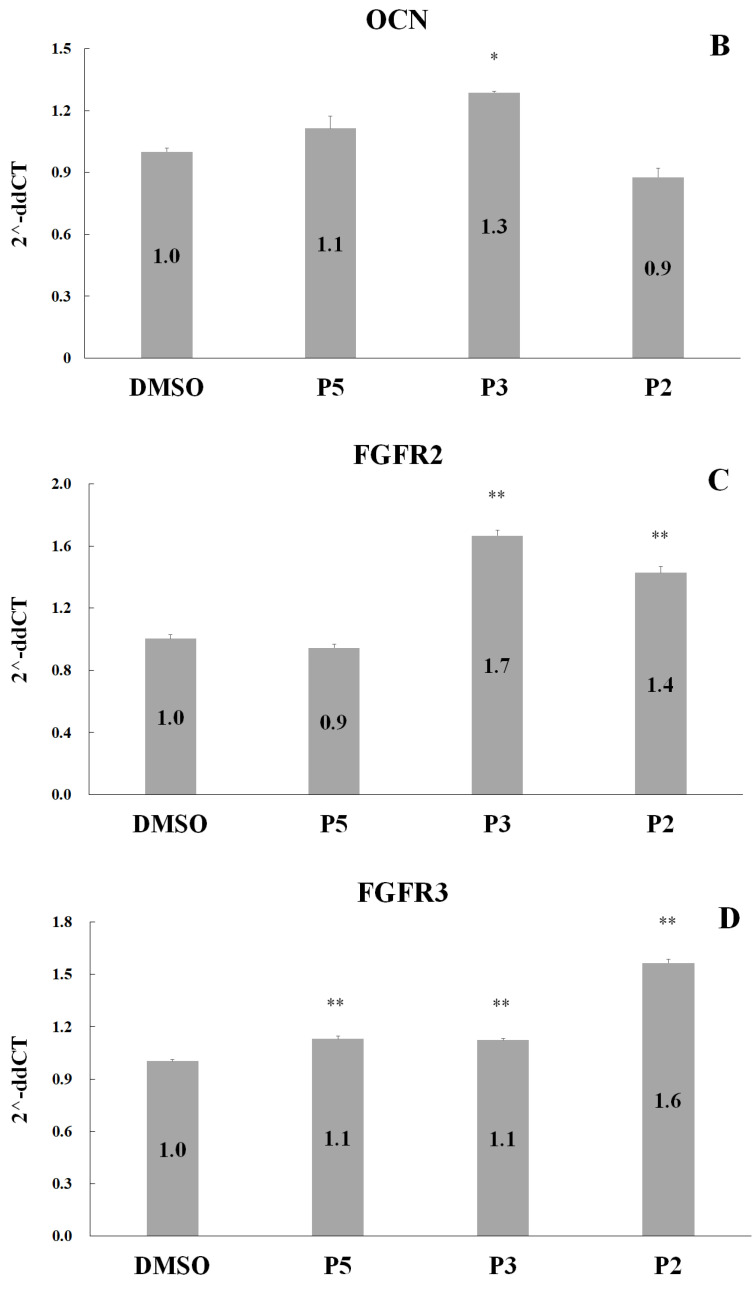

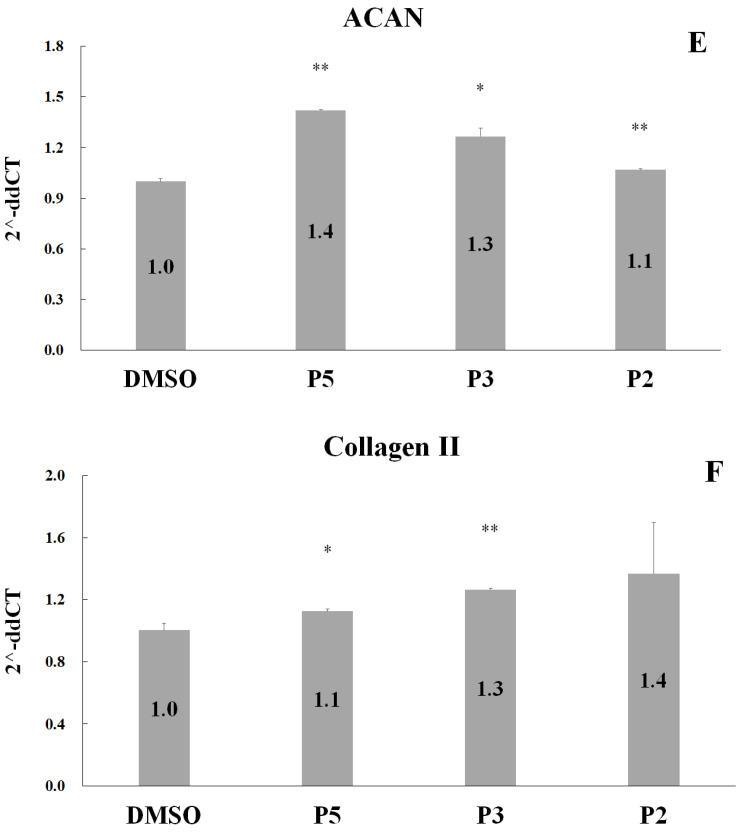
Quantitative PCR for gene expression in osteoblast hFOB1.19 and chondrocyte C20A4 cells after treatment of peptides. Cells were treated with 0.9 µM P5, 0.45 µM P3, and 0.45 µM P2 with or without the treatment of 18.4 µM LY364947/PQ401 for 2 h (**A**, RUNX2, and **B**, OCN), 6 h (**C**, FGFR2, and **D**, FGFR3), and 24 h (**E**, ACAN, and **F**, COL2A1). The differences between the various peptide treatment and the no-treatment groups (**A**)/DMSO treatment (**B**–**F**) were analyzed by a one-tailed test, * *p* < 0.05 and ** *p* < 0.005.

As shown in Figure 7A, an approximate 1.5-fold increase in RUNX2 gene expression was observed following treatment with peptide 2 and peptide 3 for 2 h. This increase could be subdued by both inhibitors PQ401 and LY364947, suggesting that both peptides regulate RUNX2 gene expression via both insulin-like growth factor and TGF-β type-1 receptors. In addition, the treatment of peptide 3 for 2 h produced a 1.3-fold increase in OCN gene expression (Figure 7B). Furthermore, treatment with peptide 2 and 3 for 6 h led to a 1.4-fold and 1.7-fold increase in FGFR2 gene expression, respectively (Figure 7C). Treatment with peptide 2 could lead to a 1.6-fold increase in FGFR3 gene expression (Figure 7D). If concurrently administered, these peptides could promote osteogenesis by upregulating RUNX2, OCN, FGFR2, and FGFR3. The expression of RUNX2, a key transcription factor associated with osteoblast differentiation, was evidently regulated by both insulin-like growth factor and TGF-β signaling pathways.

The abundant type II collagen mainly forms the fibrillary framework of the extracellular matrix (ECM) in cartilage. This framework contains sufficient glycoprotein, aggrecan, and hyaluronan to provide compressive strength. It also has water-retention properties. Notably, treatment with peptide 5 and peptide 3 led to a 1.4- and 1.3-fold increase in ACAN gene expression after 24 h, respectively (Figure 7E). Finally, peptide 3 and peptide 2 resulted in a 1.3- and 1.4-fold increase in COL2A1 gene expression after 24 h, respectively (Figure 7F).

## 3. Discussions

Traditional Chinese medicine practitioners frequently prescribe “Guilu Erxian Jiao” to osteoporosis and knee joint degeneration patients. The present study is the first to provide molecular-level evidence to support the efficacy of Guilu Erxian Jiao. Searching the genome database for red deer (*Cervus elaphus*) showed that the peptide sequence TSKYR appears in the following proteins: hemoglobin subunit alpha (Accession number: XP_043771628), katanin p60 ATPase-containing subunit A1 (Accession number: XP_043744346), katanin p60 ATPase-containing subunit A-like 1 isoform X1 (Accession number: XP_043747873), katanin p60 ATPase-containing subunit A-like 1 isoform X2 (Accession number: XP_043747878), zinc finger protein 131 isoform X1 (Accession number: XP_043743469), zinc finger protein 131 isoform X1 (Accession number: XP_043743469), 60S ribosomal protein L5-like (Accession number: XP_043726464), and piggyBac transposable element-derived protein 1 (Accession number: XP_043764083). Notably, in Chinese society, “deer blood wine” has long been thought to strengthen and replenish the body. Since the hemoglobin subunit alpha of red deer also contains this peptide sequence, we can reasonably infer that its strengthening effect could be associated with this peptide.

Notably, both kyotorphin YR and neokyotorphin TSKYR are analgesic peptides isolated from bovine brain, as reported about 45 years ago [21]. The analgesic action of these peptides was confirmed in a previous animal study using intracisternal injection [22,23]. Not only can kyotorphin be directly synthesized by a specific kyotorphin synthetase [24]; it is also able to induce an influx of Ca^+2^ from the extracellular space into the cytosol [25]. In accordance with this assumption, the strength of the proliferation-stimulating effect on osteoblast in the presence of Ca^+2^ was in the order of peptide 2 (kyotorphin) > peptide 5 (neokyotorphin) > peptide 3 > peptide 4. It seems likely that the peptides derived from “Guilu Erxian Jiao” enhance the calcium bioavailability for human bone systems.

Recent studies have shown that “tortoiseshell and deer antler gelatin” can lead to the enhancement of insulin-like growth factor levels in serum and the proliferation of osteoblasts [26]. In the glucocorticoid-induced osteoporosis zebrafish model, this Traditional Chinese Medicine can promote collagen synthesis through the TGF-β/Smad3 signal pathway and inhibit inflammatory reactions via NF-κB and AP-1 pathways [27]. In accordance with our results, as shown in Figure 7A, the upregulation of RUNX2 by these peptides in human osteoblasts is associated with both insulin-like growth factor and TGF-β receptor signaling pathways.

## 4. Materials and Methods

### 4.1. Materials

The tortoiseshell and deer antler gelatin was a product of Chuan Feng Tang Pharmaceutical Co., Ltd. (Tao-Yuan County, Taiwan) (batch number: R029901). Pepsin (catalog number: P7000) was purchased from Sigma-Aldrich (St. Louis, MO, USA). Formic acid was purchased from Honeywell (Charlotte, NC, USA). Acetonitrile was purchased from Spectrum Chemical (New Brunswick, NJ, USA). Synthetic peptides were provided by Mission Biotech Co., Ltd. (Taipei, Taiwan).

### 4.2. Separation of Bioactive Peptides by Reverse-Phase High-Performance Liquid Chromatography (HPLC)

Raw gelatin blocks were cut into small pieces using a knife. A 1 g slice of gelatin was dissolved in 10 mL water. The solution was incubated in a 100 °C water bath for 1 h, with intermittent stirring until it was homogenous. After cooling to room temperature, the pH of the solution was adjusted to 1.8 by adding 1N HCl. Ten units of pepsin protease were then added to the solution and incubated in a 37 °C water bath for 1 h. To quench the proteolytic reaction, the pH of the solution was adjusted to 7.0 by adding 1N NaOH. The 2 mL pepsin-digested samples were then filtered through a 0.22 µm syringe filter and injected directly into the HPLC column for separation and activity analysis.

All experiments separating digested peptides were conducted using a Hitachi L-7000 HPLC system (Hitachi High-Tech Corporation, Tokyo, Japan), equipped with a quaternary gradient pump (L-7100) and a photo diode array detector (L-7450). Chromatographic data were acquired and processed by Hitachi HSM software. The µBondapak^TM^ C18 Column, 125 Å, 10 µm, 3.9 × 300 mm, (Waters Corporation, Milford, MA, USA) analytical column was used. The separation was performed at room temperature.

The pepsin-digested sample was separated using a gradient elution of solvent A (0.1% formic acid) and solvent B (90% CH_3_CN, containing 0.1% formic acid) at a flow rate of 1 mL/minute. The UV detection wavelengths were 210, 254, and 280 nm. During HPLC separation, 1 mL eluent solvent was collected for each fraction. The active fractions (0–22 min) were collected, combined, and then dried via lyophilization. The dried powders collected from the four HPLC separations mentioned above were pooled together and dissolved in water to a final volume of 2 mL. This sample was then subjected to LC-MS/MS analysis. The HPLC separations were carried out at a 1 mL/minute flow rate using the HPLC elution programs in Table 1. The HPLC solvents used were as follows: solvent A (water), solvent B (water containing 0.1% formic acid), and solvent C (90% CH_3_CN, containing 0.1% formic acid) [28].

### 4.3. Identification of Bioactive Peptides by Liquid Chromatography–Mass Spectrometry (LC-MS)

LC-MS/MS experiments were performed using a Q Exactive^TM^ mass spectrometer (ThermoFisher Scientific, USA) in positive electrospray ionization mode [29]. HPLC separation conditions were described in Section 2.2. The peptide was identified in the smoothed base peak chromatograms (BPCs) using Proteome Discoverer 1.4 software (ThermoFisher Scientific, Waltham, MA, USA). The database search parameters were as follows: Type of Search—MS/MS Ion search; Enzyme—None; Variable Modifications—Deamidated (NQ), Oxidation (M); Mass Values—Monoisotopic; Protein Mass - Unrestricted; Peptide Mass Tolerance—±10 ppm; Fragment Mass Tolerance—±0.05 Da; Max Missed Cleavages—0. Bioactive peptides were identified using a UniProt (https://www.uniprot.org/, accessed on 22 June 2022) *Cervus elaphus* (red deer) protein database, which contains 20,274 sequences and 7,175,222 residues.

### 4.4. Cell Culture

Human osteoblast hFOB1.19 was purchased from the American Type Cell Collection (ATCC). This cell line was cultured in a 1:1 mixture of DMEM/F-12 medium (Dulbecco’s Modified Eagle’s Medium/Ham’s F12 Medium) containing 2.5 mM L-glutamine (without phenol red), 0.3 mg/mL G418, and 10% FBS. Cells were incubated at 34 °C in a 5% CO_2_ atmosphere [30]. Human articular chondrocyte C20A4 was obtained from MERCK (Darmstadt, Germany). The cells were cultured in DMEM high glucose medium containing 10% FBS at 37 °C in a 5% CO_2_ atmosphere [31].

### 4.5. Measurement of Cell Proliferation

To evaluate the cell proliferation activity of the four synthetic peptides, hFOB1.19 and C20A4 cells were cultured separately in 24-well plates, with 4 × 10^4^ cells per well. Samples were treated with one of several peptides at a predetermined concentration for 24 h, in the presence or absence of CaCl_2_. Cells were then treated with trypsin for 2 min and counted manually using a hemocytometer [32].

### 4.6. Alkaline Phosphatase Activity Assay (ALP)

ALP activity was measured using the TRACP & ALP Assay Kit from Takara Bio Inc. (Shiga, Japan) [33]. The hFOB1.19 osteoblast cells were cultured in a 96-well plate, with 2 × 10^4^ cells per well, for 2 d. Following the removal of culture supernatant, the cells were resuspended in normal saline. All experimental steps were carefully followed per the manufacturer’s instructions. A total of 50 µL of extraction solution was added to each well and incubated for 5 min at room temperature. Subsequently, 50 µL of substrate solution was added to each well and incubated for an additional 60 min at 37 °C. Finally, 50 µL of 0.5 N NaOH was added to each well to discontinue the reaction. After color formation, the absorbance of the solution was measured at 405 nm.

### 4.7. Quantification of Bone Nodules

Osteoblast hFOB1.19 cells were cultured in 6-well plates and medium containing 10 nM dexamethasone, 50 µg/mL ascorbic acid, 10 mM β-glycerophosphate, and peptides as indicated [34]. After incubation for 21 d, cells were fixed with 4% paraformaldehyde and stained with 5% AgNO_3_ for 30 min in a darkroom. Cells were then rinsed with distilled water and exposed to ultraviolet radiation in laminar flow for 1 h. After incubation with 5% sodium thiosulfate for 2 min, sodium thiosulfate was removed, and 0.1% nuclear fast red-aluminum sulfate solution was subsequently added. The dye was absorbed for 5 min; then, mineralized nodules were observed and photographed under a microscope. ImageJ software was utilized to quantify the average area and density of all calcified nodules. All bone nodules in each well were manually counted one by one.

### 4.8. Reverse Transcription and Quantitative Polymerase Chain Reaction

For hFOB1.19 osteoblast and chondrocyte C20A4, cells were treated with the optimal concentration of various peptides and incubated. Insulin-like growth factor receptor (IGF1R) inhibitor, PQ401, and inhibitor of TGF-β type-I receptor, LY364947, were purchased from SigmaAldrich. RNA isolation and reverse transcription were completed using a Gene-spin Total RNA purification and MMLV Reverse Transcription Kit from Protech Technology Enterprise Co., Ltd. (Taipei, Taiwan). All experimental steps were carefully followed per the manufacturer’s instructions. Primer sequences are listed in Table 2.

## 5. Conclusions

The active peptides used to stimulate the proliferation of human osteoblast and chondrocyte cells in deer antler were isolated, identified, and characterized here. We determined that including tortoiseshell considerably enhanced the content of calcium ions in this gelatin product. Perhaps unsurprisingly, synergistic effects on osteoblast proliferation and differentiation between calcium ions and these peptides were observed. Finally, we can optimistically expect that taking folk medicine or foods containing those peptides can considerably improve osteoporosis based on the results presented in this study.

## Figures and Tables

**Table 1:** HPLC elution schedules for the pepsin-digested samples.

First Separation		Second Separation for LC-MS Analysis		
Time (min)	Eluent (B%)	Time (min)	Eluent (B%)	Eluent (C%)
0–15	0	0–10	0	0
15–20	0–15	10–25	0–100	0
20–40	15	25–30	100	80–100
40–45	15–20	30–40	100–0	0–100
45–75	20	40–45		100
75–80	20–100	45–60		100–0
80–90	100			
90–100	100–0			

**Table 2:** Primer sequences for RT-qPCR of target genes.

Gene	Primer Sequence	Reference
Runx2	FORWARD: TACTTCGTCAGCATCCTATCA REVERSE: TTCCGTCAGCGTCAACAC	[13]
OCN	FORWARD: GAGGGCAATAAGGTAGTGAA REVERSE: CATAGATGCGTTTGTAGGC	[13]
FGFR2	FORWARD: GAGAAGGAGATCACGGCTTC REVERSE: AAGTCTGGCTTCTTGGTCGT	[17,18,19]
FGFR3	FORWARD: GCCTCCTCGGAGTCCTTG REVERSE: GCCTCCTCGGAGTCCTTG	[35]
ACAN	FORWARD: CACCTCCCCAACAGATGCTT REVERSE: GGTACTTGTTCCAGCCCTCC	[36]
COL2A1	FORWARD: TGCTGCCCAGATGGCTGGAGGA REVERSE: TGCCTTGAAATCCTTGAGGCCC	[37]
β-actin	FORWARD: AGAGCTACGAGCTGCCTGAC REVERSE: AGCACTGTGTTGGCGTACA	[38]

## Data Availability

Data will be made available on request.
